# Wheat *Susceptibility* Genes *TaCAMTA2* and *TaCAMTA3* Negatively Regulate Post-Penetration Resistance against *Blumeria graminis forma specialis tritici*

**DOI:** 10.3390/ijms241210224

**Published:** 2023-06-16

**Authors:** Mengmeng Li, Zige Yang, Jiao Liu, Cheng Chang

**Affiliations:** College of Life Sciences, Qingdao University, Qingdao 266071, China

**Keywords:** wheat, CAMTA transcription factor, *Blumeria graminis forma specialis tritici*, *SARD1*, *EDS1*

## Abstract

*Blumeria graminis forma specialis tritici* (*B.g. tritici*) is the airborne fungal pathogen that causes powdery mildew disease on hexaploid bread wheat. Calmodulin-binding transcription activators (CAMTAs) regulate plant responses to environments, but their potential functions in the regulation of wheat–*B.g. tritici* interaction remain unknown. In this study, the wheat CAMTA transcription factors TaCAMTA2 and TaCAMTA3 were identified as suppressors of wheat post-penetration resistance against powdery mildew. Transient overexpression of *TaCAMTA2* and *TaCAMTA3* enhanced the post-penetration susceptibility of wheat to *B.g. tritici*, while knockdown of *TaCAMTA2* and *TaCAMTA3* expression using transient- or virus-induced gene silencing compromised wheat post-penetration susceptibility to *B.g. tritici.* In addition, *TaSARD1* and *TaEDS1* were characterized as positive regulators of wheat post-penetration resistance against powdery mildew. Overexpressing *TaSARD1* and *TaEDS1* confers wheat post-penetration resistance against *B.g. tritici*, while silencing *TaSARD1* and *TaEDS1* enhances wheat post-penetration susceptibility to *B.g. tritici.* Importantly, we showed that expressions of *TaSARD1* and *TaEDS1* were potentiated by silencing of *TaCAMTA2* and *TaCAMTA3.* Collectively, these results implicated that the *Susceptibility* genes *TaCAMTA2* and *TaCAMTA3* contribute to the wheat–*B.g. tritici* compatibility might via negative regulation of *TaSARD1* and *TaEDS1* expression.

## 1. Introduction

As one of the most widely grown small-grain cereal crops, bread wheat (*Triticum aestivum* L.) has served as a major staple food for thousands of years and provided about 20% of the calories consumed by humans [1]. With the increase in the global population, the demand for wheat grains is rapidly growing [1]. However, wheat production is seriously threatened by attacks from adapted pathogens and pests [2]. Powdery mildew is a devastating disease of wheat that is caused by the obligate biotrophic fungal pathogen *Blumeria graminis forma specialis tritici* (*B.g. tritici*), leading to 5–50% yield losses [3,4]. To date, the safest, most economical, and most effective strategy to control this epidemic is breeding *B.g. tritici*-resistant wheat cultivars [3,4]. Therefore, it is critical to elucidate the molecular interaction between wheat and *B.g. tritici* and identify key regulators of wheat resistance against powdery mildew disease.

In general, plants employ two classes of immune receptors to detect adapted pathogens and initiate defense responses [5,6,7]. The pattern recognition receptors (PRRs) residing on the plant cell surface recognize the conserved pathogen-associated molecular pattern (PAMP) to initiate PAMP-triggered immunity (PTI) [8,9,10,11,12]. Upon detection of pathogen effectors, plant resistance proteins activate effector-triggered immunity (ETI) [13,14,15,16]. Although PTI and ETI are activated by distinct immune receptors and display different amplitudes and durations, they are both associated with massive transcriptomic reprogramming governed by transcription factors [17,18].

As Ca^2+^-loaded calmodulin binding (CaMB) transcription factors, calmodulin-binding transcription activators (CAMTAs) play important roles in regulating plant growth, development, and responses to environmental stresses [19,20,21]. For instance, expressions of six *CAMTA* genes differentially respond to environmental cues like drought, salinity, and extreme temperatures in the model plant *Arabidopsis thaliana* [22,23,24,25,26]. *Arabidopsis* mutant *camta1* exhibited hypersensitivity to cold and drought stress, and AtCAMTA1 was shown to regulate the expression of cold and drought-responsive genes like *AtRD26*, *AtERD7*, *AtCBF2*, and *AtRAB18* [22,23,24,25,26]. 4- to 11-day-old *Arabidopsis* mutant *camta6* exhibited hypersensitivity to NaCl treatment, and AtCAMTA6 was demonstrated to regulate expression of salt resilience-related genes, including *HIGH-AFFINITY K^+^ TRANSPORTER1*, *SALT OVERLY SENSITIVE1*, and *Na^+^/H^+^ ANTIPORTER* [27]. In addition, CAMTA transcription factors get involved in the regulation of plant defense against pathogens. For instance, *Arabidopsis* AtCAMTA3 was shown to function in concert with AtCAMTA1 and AtCAMTA2 in suppressing plant defense responses [28,29,30,31,32]. However, whether and how CAMTA transcription factors regulate wheat disease resistance against *B.g. tritici* remains largely unknown.

In this research, two CAMTA transcription factor genes, *TaCAMTA2* and *TaCAMTA3*, were characterized as *Susceptibility* (*S*) genes contributing to wheat–*B.g. tritici* compatibility. Transient overexpression of *TaCAMTA2* and *TaCAMTA3* resulted in enhanced wheat post-penetration susceptibility to *B.g. tritici*, while transient silencing of *TaCAMTA2* and *TaCAMTA3* led to attenuated wheat post-penetration susceptibility to *B.g. tritici.* Furthermore, overexpressing *TaSARD1* and *TaEDS1* could confer wheat post-penetration resistance against powdery mildew, while silencing *TaSARD1* and *TaEDS1* enhanced wheat post-penetration susceptibility to *B.g. tritici*. Moreover, *TaCAMTA2* and *TaCAMTA3* were demonstrated to negatively regulate the expression of the defense genes *TaSARD1* and *TaEDS1.* These results strongly support that *S* genes *TaCAMTA2* and *TaCAMTA3* partially redundantly suppress wheat post-penetration resistance against *B.g. tritici* presumably via the negative regulation of expressions of defense genes *TaSARD1* and *TaEDS1*.

## 2. Results

### 2.1. Homology-Based Identification of TaCAMAT2 and TaCAMTA3 in Bread Wheat

Previous studies revealed that the *Arabidopsis* CAMTA transcription factor AtCAMTA3 plays a vital role in the regulation of plant immunity [29,30,31,32]. In this study, we are interested in exploring the function of the wheat homolog of AtCAMTA3 in the wheat–*B.g. tritici* interaction. To this end, we first searched the reference genome of the hexaploid bread wheat by using the amino acid sequence of *Arabidopsis* AtCAMTA3 (At2g22300) as a query and obtained *TaCAMAT2* and *TaCAMTA3*, the most closely related homologs of *AtCAMTA3*, in bread wheat. Three highly homologous sequences of *TaCAMAT2* genes separately located on chromosomes 4A, 4B, and 4D were obtained from the genome sequence of the hexaploid wheat and designated as *TaCAMTA2-4A* (TraesCS4A02G407100), *TaCAMTA2-4B* (TraesCS4B02G306300), and *TaCAMTA2-4D* (TraesCS4D02G304500). Similarly, three highly homologous sequences of *TaCAMAT3* genes separately located on chromosomes 2A, 2B, and 2D were obtained from the genome sequence of the hexaploid wheat and designated as *TaCAMTA3-2A* (TraesCS2A02G163000), *TaCAMTA3-2B* (TraesCS2B02G188800), and *TaCAMTA3-2D* (TraesCS2D02G169900).

**Figure 1:**
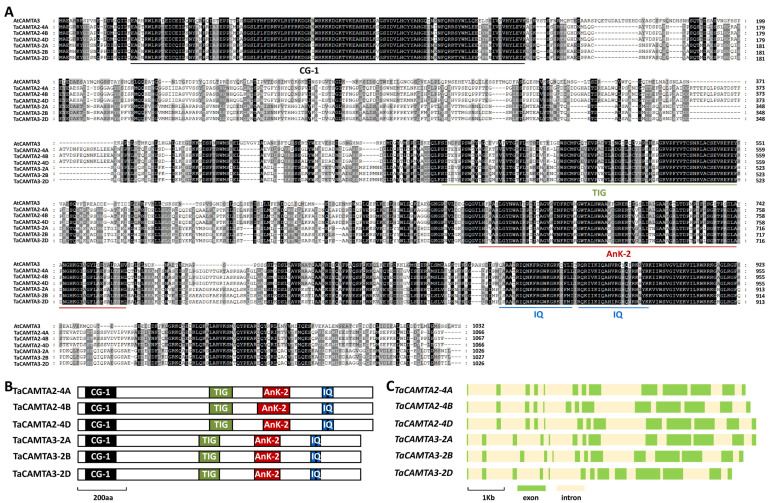
Identification of wheat TaCAMTA2 and TaCAMTA3 based on homology with *Arabidopsis* AtCAMTA3. (**A**) Protein sequence comparison of wheat TaCAMTA2, TaCAMTA3, and *Arabidopsis* AtCAMTA3. Residues conserved in at least 4 of the 7 proteins are shaded in gray, while identical residues among 7 protein sequences are shaded in dark. (**B**) Domain structure of wheat TaCAMTA2 and TaCAMTA3 proteins. (**C**) Gene architectures of the wheat *TaCAMTA2* and *TaCAMTA3* genes.

As shown in Figure 1A, these predicted TaCAMTA2-4A, TaCAMTA2-4B, TaCAMTA2-4D, TaCAMTA3-2A, TaCAMTA3-2B, and TaCAMTA3-2D proteins shared about 46% identity with *Arabidopsis* AtCAMTA3. In addition, TaCAMTA2-4A, TaCAMTA2-4B, TaCAMTA2-4D, TaCAMTA3-2A, TaCAMTA3-2B, and TaCAMTA3-2D proteins all contain a conserved CG-1 DNA-binding domain at their N-terminal parts, a transcription factor immunoglobulin-like (TIG) DNA-binding domain, several ankyrin repeats (ANK) in the middle parts, as well as two IQ CaMB motifs (IQXXXRGXXXR) at their C-termini (Figure 1B). The coding regions of these allelic *TaCAMAT2* and *TaCAMTA3* genomic sequences all contained 13 exons and 12 introns (Figure 1C).

### 2.2. TaCAMAT2 and TaCAMTA3 Contribute to the Wheat Susceptibility to B.g. tritici

**Figure 2:**
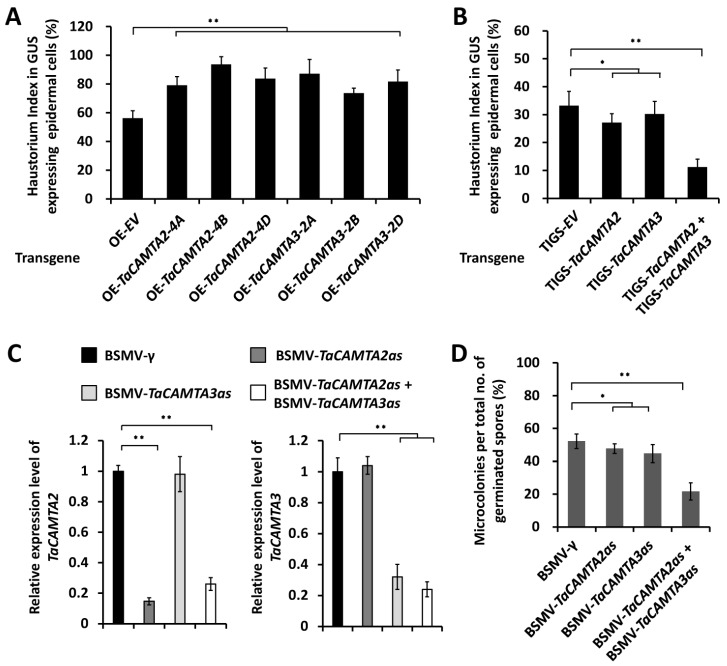
Functional analyses of wheat *TaCAMTA2* and *TaCAMTA3* under *B.g. tritici* infection. (**A**) Haustorial index analysis in wheat epidermal cells transiently overexpressing *TaCAMTA2* (*OE-TaCAMTA2*) and *TaCAMTA3* (*OE-TaCAMTA3*). Haustorial formation on wheat epidermal cells bombarded with empty vector (*OE-EV*) was statistically analyzed as a control. At least 100 wheat cells were analyzed in each experiment. (**B**) Haustorial index analysis in wheat epidermal cells transiently silencing *TaCAMTA2* (*TIGS-TaCAMTA2*), *TaCAMTA3* (*TIGS-TaCAMTA3*), or co-silencing *TaCAMTA2* and *TaCAMTA3* (*TIGS-TaCAMTA2* + *TIGS-TaCAMTA3*). Haustorial formation on wheat epidermal cells bombarded with an empty vector (*TIGS-EV*) was statistically analyzed as a control. (**C**) qRT-PCR analysis of *TaCAMTA2* and *TaCAMTA3* expression in wheat leaves infected with the indicated BSMV vectors. BSMV-γ empty vector was employed as the negative control. (**D**) *B.g. tritici* microcolony index analysis on wheat leaves silencing *TaCAMTA2* (*BSMV-TaCAMTA2as*), *TaCAMTA3* (*BSMV-TaCAMTA3as*), or co-silencing *TaCAMTA2* and *TaCAMTA3* (*BSMV-TaCAMTA2as* + *BSMV-TaCAMTA3as*). At least 1000 wheat–*B.g. tritici* interaction sites were counted in one experiment for each treatment. For (**A**–**D**), three independent biological replicates were statistically analyzed for each treatment (*t*-test; * *p* < 0.05, ** *p* < 0.01).

To study the function of *TaCAMAT2* and *TaCAMTA3* in the wheat–*B.g. tritici* interaction, we first employed transient gene expression assays to overexpress these *TaCAMTA2-4A*, *TaCAMTA2-4B*, *TaCAMTA2-4D*, *TaCAMTA3-2A*, *TaCAMTA3-2B*, or *TaCAMTA3-2D* genes in the leaf epidermal cells of the *B.g. tritici*-susceptible wheat cultivar Yannong 999. After inoculation of conidia from the virulent *B.g. tritici* isolate E09, the formation of fungal haustoria in the transformed wheat cells was statistically analyzed. As shown in Figure 2A, the *B.g. tritici* haustorium index (HI%) increased from 56% for the empty vector (OE-EV) control to above 70% on wheat cells overexpressing *TaCAMTA2* or *TaCAMTA3* genes. These results suggested that overexpression of *TaCAMAT2* and *TaCAMTA3* could significantly enhance wheat post-penetration susceptibility to *B.g. tritici*.

To further verify the function of *TaCAMAT2* and *TaCAMTA3* in the regulation of wheat–*B.g. tritici* interaction, we employed transiently induced gene silencing (TIGS) assays to silence all endogenous *TaCAMAT2* or *TaCAMTA3* genes in the epidermal cell of the *B.g. tritici*-susceptible wheat cultivar Yannong 999. After inoculation of conidia from the virulent *B.g. tritici* isolate E09, the frequency of fungal haustorium formation in the transformed plant cells was scored. As shown in Figure 2B, the silencing of *TaCAMAT2* or *TaCAMTA3* genes resulted in a marked HI% decrease to about 27%, compared to 33% for empty vector (EV) controls. Significantly, simultaneous silencing of *TaCAMAT2* and *TaCAMTA3* could lead to a further decrease in HI% to approximately 13%, suggesting that *TaCAMTA2* and *TaCAMTA3* might partially redundantly suppress post-penetration resistance of wheat to *B.g. tritici.*

In addition, we performed barley stripe mosaic virus (BSMV)-induced gene silencing (BSMV-VIGS) to silence all endogenous *TaCAMAT2* or *TaCAMTA3* genes in the leaves of the *B.g. tritici*-susceptible wheat cultivar Yannong 999. qRT-PCR showed that the endogenous transcript level of *TaCAMAT2* or *TaCAMTA3* was substantially reduced in the indicated VIGS plants (Figure 2C). Thereafter, these VIGS plants were inoculated with conidia from the virulent *B.g. tritici* isolate E09, and the formation of microcolonies was analyzed to evaluate the wheat’s susceptibility to powdery mildew. *B.g. tritici* microcolony index (MI%) declined to approximate 40% on BSMV-*TaCAMTA2as* plants and 47% on BSMV-*TaCAMTA3as* plants, compared with 55% for the BSMV-γ plants (Figure 2D). Notably, simultaneous silencing of *TaCAMAT2* and *TaCAMTA3* could lead to a further MI% decrease to about 28%. These data clearly indicate that *TaCAMAT2* and *TaCAMTA3* partially redundantly contribute to the wheat susceptibility to *B.g. tritici*.

### 2.3. Homology-Based Identification of TaSARD1 and TaEDS1 in Bread Wheat

Previous studies revealed that AtCAMTA3 could regulate the expression of defense genes *SYSTEMIC ACQUIRED RESISTANCE DEFICIENT 1* (*AtSARD1*) and *ENHANCED DISEASE SUSCEPTIBILITY 1* (*AtEDS1*) in *A. thaliana* [29,30,31,32]. We are interested in examining the potential regulation of TaCAMAT2 and TaCAMTA3 on the wheat defense genes. To this end, we first searched the reference genome of the hexaploid bread wheat by using the amino acid sequences of *Arabidopsis AtSARD1* (At1g73805) and *AtEDS1* (At3g48090) as a query and obtained *TaSARD1* and *TaEDS1*, the most closely related homologs of *AtSARD1* and *AtEDS1*, in bread wheat. Five highly homologous sequences of *TaSARD1* genes separately located on chromosomes 6A, 6B, and 6D were obtained from the genome sequence of the hexaploid wheat and designated as *TaSARD1.1-6A* (TraesCS6A02G091700), *TaSARD1.1-6B* (TraesCS6B02G119900), *TaSARD1.1-6D* (TraesCS6D02G080500), *TaSARD1.2-6A* (TraesCS6A02G296600), and *TaSARD1.2-6D* (TraesCS6D02G276800). Similarly, three highly homologous sequences of *TaEDS1* genes separately located on chromosomes 5A, 5B, and 5D were obtained from the genome sequence of the hexaploid wheat and designated as *TaEDS1-5A*, *TaEDS1-5B*, and *TaEDS1-5D* [33].

**Figure 3:**
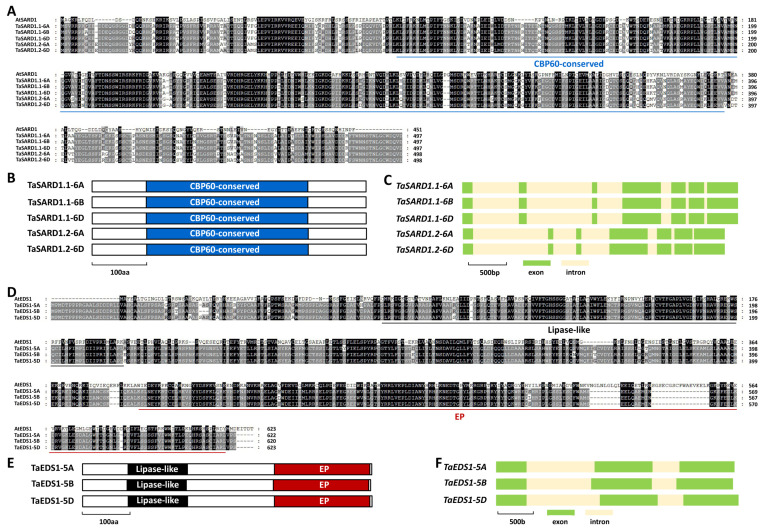
Identification of wheat TaSARD1 and TaEDS1 based on homology with *Arabidopsis* AtSARD1 and AtEDS1. (**A**) Protein sequence comparison of wheat TaSARD1 and *Arabidopsis* AtSARD1. Residues conserved in at least 3 of the 6 proteins are shaded in gray, while identical residues among 6 protein sequences are shaded in dark. (**B**) Domain structure of wheat TaSARD1 proteins. (**C**) Gene architectures of wheat *TaSARD1* genes. (**D**) Protein sequence comparison of wheat TaEDS1 and *Arabidopsis* AtEDS1. Residues conserved in at least 2 of the 4 proteins are shaded in gray, while identical residues among 4 protein sequences are shaded in dark. (**E**) Domain structure of wheat TaEDS1 proteins. (**F**) Gene architectures of wheat *TaEDS1* genes.

As shown in Figure 3A, these predicted TaSARD1.1-6A, TaSARD1.1-6B, TaSARD1.1-6D, TaSARD1.2-6A, and TaSARD1.2-6D proteins shared about 43% identities with *Arabidopsis* AtSARD1. In addition, TaSARD1.1-6A, TaSARD1.1-6B, TaSARD1.1-6D, TaSARD1.2-6A, and TaSARD1.2-6D proteins all contain a CBP60-conserved domain (Figure 3B). The coding regions of these allelic *TaSARD1* genomic sequences all contained seven exons and six introns (Figure 3C). The predicted TaEDS1-5A, TaEDS1-5B, and TaEDS1-5D proteins shared about 38% identity with *Arabidopsis* AtEDS1 (Figure 3D). In addition, TaEDS1-5A, TaEDS1-5B, and TaEDS1-5D proteins all contain an N-terminal lipase-like domain and a C-terminal EP (EDS1–PAD4) domain (Figure 3E). The coding regions of these allelic *TaEDS1* genomic sequences all contained 3 exons and 2 introns (Figure 3F).

### 2.4. TaSARD1 and TaEDS1 Positively Contribute to the Wheat Post-Penetration Resistance to B.g. tritici

**Figure 4:**
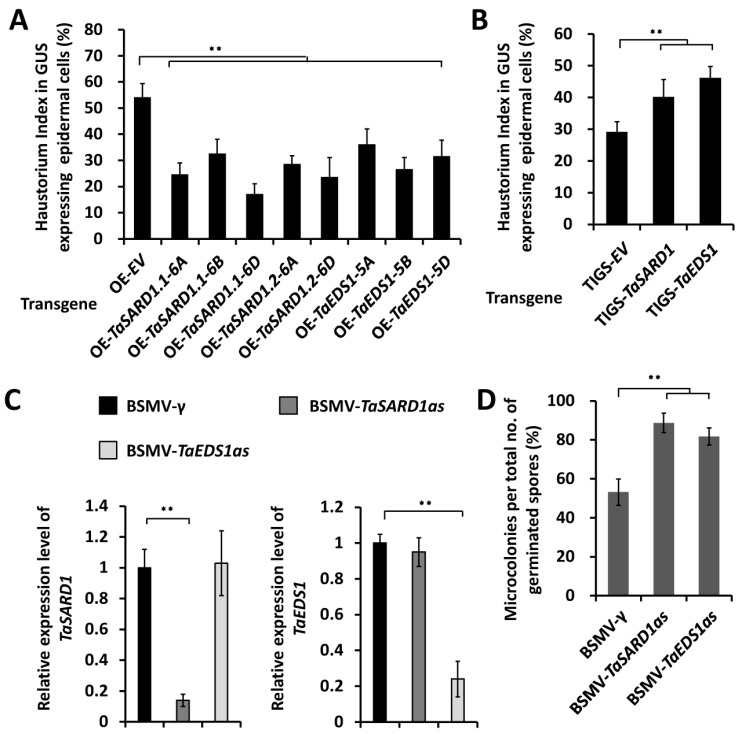
Functional analyses of wheat *TaSARD1* and *TaEDS1* under *B.g. tritici* infection. (**A**) Haustorial index analysis in wheat epidermal cells transiently overexpressing *TaSARD1* (*OE-TaSARD1*) and *TaEDS1* (*OE-TaEDS1*). Haustorial formation on wheat epidermal cells bombarded with empty vector (*OE-EV*) was statistically analyzed as a control. At least 100 wheat cells were analyzed in each experiment. (**B**) Haustorial index analysis in wheat epidermal cells transiently silencing *TaSARD1* (*TIGS-TaSARD1*) or *TaEDS1* (*TIGS-TaEDS1*). Haustorial formation on wheat epidermal cells bombarded with an empty vector (*TIGS-EV*) was statistically analyzed as a control. (**C**) qRT-PCR analysis of *TaSARD1* and *TaEDS1* expression in wheat leaves infected with the indicated BSMV vectors. The BSMV-γ empty vector was employed as the negative control. (**D**) *B.g. tritici* microcolony index analysis on wheat leaves silencing *TaSARD1* (*BSMV-TaSARD1as*) or *TaEDS1* (*BSMV-TaEDS1as*). At least 1000 wheat–*B.g. tritici* interaction sites were counted in one experiment for each treatment. For (**A**–**D**), three independent biological replicates were statistically analyzed for each treatment (*t*-test; ** *p* < 0.01).

To characterize the function of *TaSARD1* and *TaEDS1* in the wheat–*B.g. tritici* interaction, we first employed transient gene expression assays to overexpress *TaSARD1.1-6A*, *TaSARD1.1-6B*, *TaSARD1.1-6D*, *TaSARD1.2-6A*, *TaSARD1.2-6D*, *TaEDS1-5A*, *TaEDS1-5B*, or *TaEDS1-5D* genes in the leaf epidermal cell of the *B.g. tritici*-susceptible wheat cultivar Yannong 999. As shown in Figure 4A, the *B.g. tritici* HI% decreased from 54% for the empty vector control to less than 41% on wheat cells overexpressing *TaSARD1* or *TaEDS1* genes. These results suggested that overexpression of *TaSARD1* or *TaEDS1* remarkably attenuated wheat post-penetration susceptibility to *B.g. tritici*.

To further examine the function of *TaSARD1* and *TaEDS1* in regulating wheat–*B.g. tritici* interaction, we employed the TIGS assays to silence all endogenous *TaSARD1* or *TaEDS1* genes in the leaf epidermal cell of the *B.g. tritici*-susceptible wheat cultivar Yannong 999. As shown in Figure 4B, silencing of *TaSARD1* or *TaEDS1* genes resulted in a notable HI% increase to above 42%, compared to 31% for empty vector controls. In addition, we employed BSMV-VIGS to silence all endogenous *TaSARD1* or *TaEDS1* genes in the leaves of the *B.g. tritici*-susceptible wheat cultivar Yannong 999. qRT-PCR showed that the endogenous transcript level of *TaSARD1* or *TaEDS1* was significantly reduced in the indicated VIGS plants (Figure 4C). Thereafter, these VIGS plants were inoculated with *B.g. tritici* conidia, and the formation of microcolonies was statistically analyzed. *B.g. tritici* MI% increased to approximately 65% on BSMV-*TaSARD1as* plants and 72% on BSMV-*TaEDS1as* plants, compared with 53% for the BSMV-γ plants (Figure 4D). These data support that *TaSARD1* and *TaEDS1* positively regulate the wheat post-penetration resistance to *B.g. tritici.*

### 2.5. TaCAMAT2 and TaCAMTA3 Negatively Regulate Expression of TaSARD1 and TaEDS1

**Figure 5:**
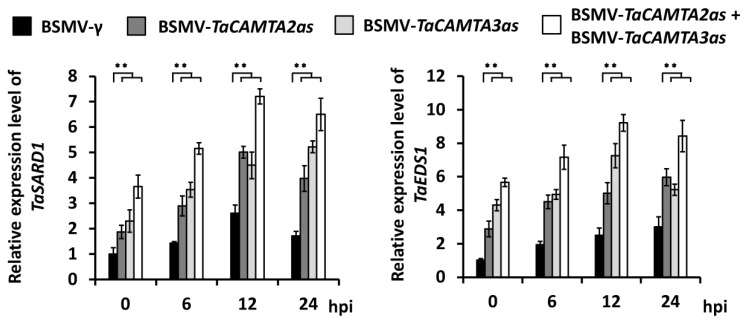
qRT-PCR analysis of *TaSARD1* and *TaEDS1* expression levels in *TaCAMTA2* and *TaCAMTA3* silenced wheat leaves under *B.g. tritici* infection. The data are shown as means ± SEs (*t*-test; ** *p* < 0.01) from three independent biological replicates. hpi is the abbreviation for hours post *B.g. tritici* inoculation.

To determine the potential regulation of *TaCAMAT2* and *TaCAMTA3* on the expression of *TaSARD1* and *TaEDS1* in bread wheat, we employed BSMV-VIGS to silence all endogenous *TaCAMAT2* or *TaCAMTA3* genes in the leaves of the *B.g. tritici*-susceptible wheat cultivar Yannong 999. Thereafter, these VIGS plants were inoculated with *B.g. tritici* conidia, and expression levels of *TaSARD1* and *TaEDS1* were analyzed. As shown in Figure 5, the silencing of *TaCAMAT2* or *TaCAMTA3* genes resulted in a marked increase in the expression levels of *TaSARD1* and *TaEDS1.* Significantly, simultaneous silencing of *TaCAMAT2* and *TaCAMTA3* could lead to a further increase in the expression levels of *TaSARD1* and *TaEDS1*, suggesting that partially redundant *TaCAMTA2* and *TaCAMTA3* negatively regulate the expressions of *TaSARD1* and *TaEDS1.*

**Figure 6:**
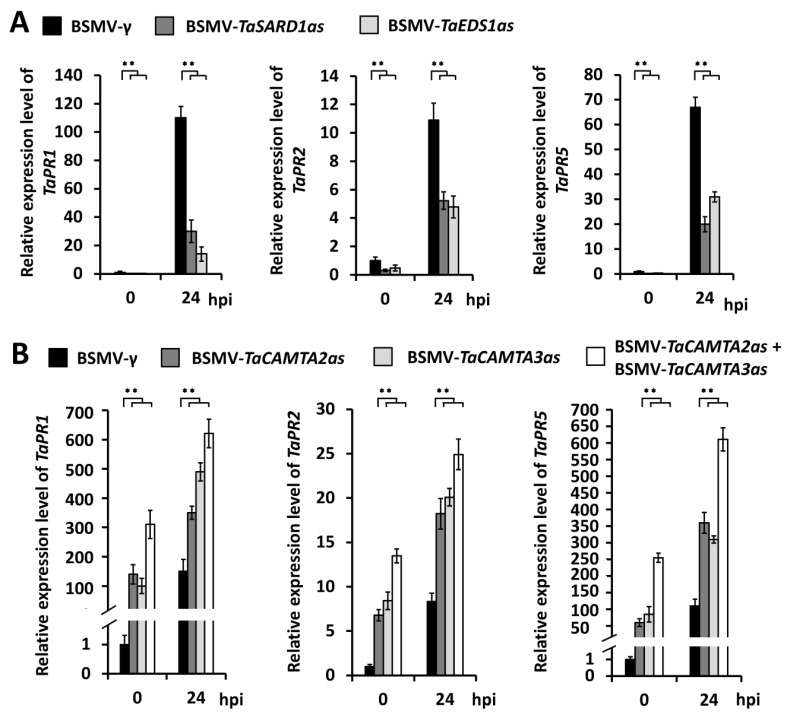
*TaPR1*, *TaPR2*, and *TaPR5* expression levels in BSMV-VIGS wheat leaves. (**A**) qRT-PCR analysis of *TaPR1*, *TaPR2*, and *TaPR5* expression levels in *TaSARD1* and *TaEDS1* silenced wheat leaves under *B.g. tritici* infection. (**B**) RT-PCR analysis of *TaPR1*, *TaPR2*, and *TaPR5* expression levels in *TaCAMTA2* and *TaCAMTA3* silenced wheat leaves under *B.g. tritici* infection. The data are shown as means ± SEs (*t*-test; ** *p* < 0.01) from three independent biological replicates.

Since *PR* expressions are usually activated in the plant defense responses to biotrophic pathogens like *B.g. tritici*, we compared the transcript levels of *TaPR1*, *TaPR2*, and *TaPR5* among BSMV-*TaCAMTA2*as, BSMV-*TaCAMTA3*as, BSMV-*TaSARD1*as, BSMV-*TaEDS1*as, and BSMV-γ infected plants. As shown in Figure 6A, the expressions of *TaPR1*, *TaPR2*, and *TaPR5* were remarkably reduced by silencing of *TaSARD1* or *TaEDS1*, further confirming the fact that *TaSARD1* and *TaEDS1* positively regulate the wheat defense against *B.g. tritici*. In contrast, the expressions of *TaPR1*, *TaPR2*, and *TaPR5* were significantly affected by the silencing of *TaCAMAT2* or *TaCAMTA3* genes (Figure 6B). Notably, simultaneous silencing of *TaCAMAT2* and *TaCAMTA3* could lead to a further increase in the activation of *TaPR1*, *TaPR2*, and *TaPR5* (Figure 6B), which is consistent with the fact that partially redundant *TaCAMTA2* and *TaCAMTA3* negatively regulate expressions of the wheat defense genes *TaSARD1* and *TaEDS1.*

## 3. Discussion

### 3.1. TaCAMAT2 and TaCAMTA3 Are Wheat S Genes Suppressing Post-Penetration Resistance against B.g. tritici

Powdery mildew, caused by the adapted fungal pathogen *B.g. tritici*, seriously threatens global wheat production [3,4]. To improve wheat resistance against powdery mildew, it is vital to identify the important genes involved in the regulation of the wheat–*B.g. tritici* interaction [3,4]. *Powdery mildew* (*Pm*) resistance genes and *quantitative trait loci* (*QTL*) contributed to wheat resistance to *B.g. tritici* and have been employed in wheat breeding for powdery mildew resistance [3,4]. Compatibility between wheat and *B.g. tritici* underlies wheat’s susceptibility to powdery mildew. A plethora of wheat *S* genes have been identified to facilitate compatibility by inducing *B.g. tritici* (pre)penetration, suppressing wheat immunity, and supporting the sustenance of *B.g. tritici* [34,35]. For instance, wheat *S* genes *TaWIN1*, *TaKCS6*, and *TaECR* were revealed to facilitate the conidial germination of *B.g. tritici* by promoting the biosynthesis of wheat cuticular wax, whereas wheat *S* gene *TaSTP13* encodes a sugar transporter facilitating wheat hexose accumulation for *B.g. tritici* acquisition [36,37,38,39,40,41]. *TaMLO*, *TaEDR1*, and *TaPOD70* genes contribute to wheat susceptibility to powdery mildew by suppressing plant defense responses [42,43,44,45,46,47]. In addition, S factors TaMED25, TaHDA6, TaHOS15, and TaHDT701 positively contribute to wheat susceptibility to *B.g. tritici* by suppressing defense-related transcriptional reprogramming in bread wheat [48,49,50,51,52,53].

Through homology-based searching, *TaCAMAT2* and *TaCAMTA3* were identified as the most closely related homologs of *AtCAMTA3*, which is consistent with the reported phylogenetic analysis of the CAMTA homologs in different species [19]. *TaCAMAT2* and *TaCAMTA3* are characterized as wheat *S* genes contributing to the wheat post-penetration susceptibility to *B.g. tritici* in this study. Overexpression of *TaCAMTA2* and *TaCAMTA3* in the leaf epidermal cell by transient gene expression assays led to enhanced wheat susceptibility to *B.g. tritici*, while knockdown of *TaCAMTA2* and *TaCAMTA3* expression using transient- or virus-induced gene silencing resulted in compromised wheat post-penetration susceptibility to *B.g. tritici.* Interestingly, a gain-of-function mutation in *SIGNAL RESPONSIVE1* (*SR1*), which encodes the *Arabidopsis* homologs of wheat *TaCAMTA2* and *TaCAMTA3*, could suppress the *edr2*-associated powdery mildew resistance [29]. The *sr1-4D* single mutant is more susceptible to *Arabidopsis* powdery mildew (*Golovinomyces cichoracearum*), whereas the *sr1-1* null mutant plants displayed enhanced post-penetration resistance against *G. cichoracearum* [29]. In addition, *Arabidopsis AtCAMTA1* was revealed to function partially redundantly with *AtCAMTA2* and *AtCAMTA3* in suppressing plant immunity [30,31,32]. In this study, simultaneous silencing of *TaCAMAT2* and *TaCAMTA3* could lead to a further decrease in the HI% and MI% compared with single silencing of *TaCAMAT2* or *TaCAMTA3*, supporting the fact that *TaCAMTA3* functions partially redundantly with *TaCAMAT2* in suppressing wheat post-penetration resistance against *B.g. tritici*. In *Arabidopsis*, CAMTA transcription factors AtCAMTA1, AtCAMTA2, and AtCAMTA3 partially redundantly suppress the biosynthesis of salicylic acid (SA) and N-hydroxypipecolic acid (NHP), a metabolite duo essential for systemic acquired resistance (SAR) [30,31,32]. Therefore, it is intriguing to examine the potential roles of the *S* genes *TaCAMAT2* and *TaCAMTA3* in the regulation of SA and NHP biosynthesis, as well as SAR establishment, in bread wheat in future research.

### 3.2. TaSARD1 and TaEDS1 Confer Wheat Post-Penetration Resistance against B.g. tritici

*TaSARD1* and *TaEDS1* are identified as positive regulators of wheat resistance against *B.g. tritici* in this study. Overexpression of *TaSARD1* or *TaEDS1* in the leaf epidermal cell by transient gene expression assays led to enhanced wheat post-penetration resistance to *B.g. tritici*, while knockdown of *TaSARD1* or *TaEDS1* expression using transient- or virus-induced gene silencing resulted in increased wheat post-penetration susceptibility to *B.g. tritici.* In *Arabidopsis*, transcription factor AtSARD1 functions in concert with AtCBP60g to activate the expression of *SID2* (*SA INDUCTION DEFICIENT 2*), which encodes isochorismate synthase 1 (ICS1), essential for pathogen-induced SA biosynthesis [54,55,56]. *Arabidopsis* AtEDS1 was shown to heterodimerize with its partners, phytoalexin deficient 4 (PAD4) or senescence-associated gene 101 (SAG101), to play signaling roles in ETI as well as SA-dependent and SA-independent PTI pathways [57,58,59,60,61,62,63,64]. Consistent with this, expressions of SA defense marker genes *TaPR1*, *TaPR2*, and *TaPR5* induced by *B.g. tritici* infection were attenuated by silencing of *TaSARD1* or *TaEDS1*, suggesting that the *SARD1*-*EDS1*-SA defense axis might be partially conserved between model plant *Arabidopsis* and crop plant bread wheat. Therefore, it is intriguing to examine the potential regulation of wheat SA biosynthesis and signaling by *TaSARD1* and *TaEDS1* in future research.

### 3.3. TaCAMAT2 and TaCAMTA3 Negatively Regulate the Expression of TaSARD1 and TaEDS1 to Suppress Wheat Post-Penetration Resistance against B.g. tritici

In this study, expression levels of *TaSARD1* and *TaEDS1* were significantly enhanced by silencing *TaCAMTA2* and *TaCAMTA3*. Notably, simultaneous silencing *TaCAMAT2* and *TaCAMTA3* could lead to a further increase in the expression levels of *TaSARD1* and *TaEDS1* compared with single silencing *TaCAMAT2* or *TaCAMTA3*, indicating that *TaCAMTA2* and *TaCAMTA3* partially redundantly suppress expressions of *TaSARD1* and *TaEDS1.* In *Arabidopsis*, AtCAMTA3 could bind to the promoter region of *AtEDS1* by recognizing the CGCG box, thereby directly repressing the expression of *AtEDS1* [28,29,30,31]. In addition, the expression of *AtSARD1* was demonstrated to be negatively regulated by partially redundant AtCAMTA1, AtCAMTA2, and AtCAMTA3, presumably via an indirect effect [28,29,30,31]. These results indicate that negative regulation of the expressions of defense genes *SARD1* and *EDS1* by partially redundant CAMTA3 and its homologs might be partly conserved between the model plant *Arabidopsis* and the important crop bread wheat. Indeed, the expressions of SA defense marker genes *TaPR1*, *TaPR2*, and *TaPR5* induced by *B.g. tritici* infection were found to be potentiated by silencing *TaCAMAT2* or *TaCAMTA3* in this study. However, binding sites for TaCAMAT2 and TaCAMTA3 in the promoter regions of *TaSARD1* and *TaEDS1* genes remain to be identified.

Herein, *TaCAMAT2* and *TaCAMTA3* are identified as wheat *S* genes partially redundantly suppressing post-penetration resistance against powdery mildew, presumably via negative regulation of the expressions of defense genes *TaSARD1* and *TaEDS1*. Genetic manipulation of S genes *TaMLO* and *TaEDR1* via targeting induced local lesions in genomes (TILLING) and genome editing techniques like transcription activator-like effector nucleases (TALENs) and clustered regularly interspaced short palindromic repeats (CRISPR)-Cas (CRISPR-associated) 9 systems compromised wheat compatibility with *B.g. tritici* and conferred wheat resistance against powdery mildew [65,66,67,68,69,70,71,72,73]. Therefore, it is intriguing to examine the potential of manipulating the S genes *TaCAMAT2* and *TaCAMTA3* in wheat breeding for powdery mildew resistance in future research.

## 4. Materials and Methods

### 4.1. Plant and Fungal Materials

The seedlings of bread wheat cultivar Yannong999 used in this study were grown in a growth chamber under a 16-h/8-h, 20 °C/18 °C day/night cycle with 70% relative humidity. The *B.g. tritici* strain E09 was maintained on the leaves of Jing411 plants. Conidia of *B.g. tritici* strain E09 were used for the inoculation of Jing411 leaves in the study of wheat–powdery mildew interaction. *Arabidopsis thaliana* used in this study was grown in the greenhouse under a 16 h/8 h light period at 23 ± 1 °C with 70% relative humidity.

### 4.2. Quantitative Real-Time PCR (qRT-PCR)

Total RNA was extracted from the wheat leaves using the EasyPure Plant RNA kit (Transgenbiotech, Beijing, China) and 2 μg of RNA was used to synthesize the cDNA template using the TransScript one-step gDNA removal and cDNA synthesis supermix (Transgenbiotech, Beijing, China) according to the manufacturer’s instructions. The real-time PCR assay was performed using the ABI real-time PCR system with the qPCR Master Mix (Invitrogen, Carlsbad, CA, USA). The expression of traditional housekeeping gene *GLYCERALDEHYDE-3-PHOSPHATE DEHYDROGENASE (TaGAPDH)* was set as the internal control and expressions of *TaGAPDH*, *TaCAMTA2*, *TaCAMTA3*, *TaSARD1*, *TaEDS1*, *TaPR1*, *TaPR2* and *TaPR5* were analyzed using the primers 5′-TTAGACTTGCGAAGCCAGCA-3′/5′-AAATGCCCTTGAGGTTTCCC-3′, 5′-TACAGAAGTTGCAACAG-3′/5′-ATCTCCGTCGACTCCTCA-3′, 5′-CCTGACAAACAACTTGA-3′/5′-CGCCAGCTGCA TCGCTT-3′, 5′-GCGAGTAATGAAAGCAT-3′/5′-TTAATCAACTTGATCCC-3′, 5′-TGAAAGACAGGGTGGGT-3′/5′-CGAAGGCACAAGTCTCG-3′, 5′-GAGAATGCAGACGCCCAAGC-3′/5′-CTGGAGCTTGCAGTCGTTGATC-3′, 5′-AGGATGTTGCTTCCATGTTTGCCG-3′/5′-AAGTAGATGCGCATGCCGTTGATG-3′, and 5′-CTTCTACATCAAGA ACAACTG-3′/5′-CAGTCGCCGGTCTGGCAG-3′.

### 4.3. BSMV-Mediated Gene Silencing and B.g. tritici Infection

The antisense fragment of *TaCAMTA2*, *TaCAMTA3*, *TaSARD1*, and *TaEDS1* was cloned into the pCa-γbLIC vector to create the *BSMV-TaCAMTA2as*, *BSMV-TaCAMTA3as*, *BSMV-TaSARD1as*, and *BSMV-TaEDS1as* constructs using the primer pair 5′-AAGGAAGTTTATACCATCATTAGCACTTGG-3′/5′-AACCACCACCACCGTCACTTTTGGAATTACATTC-3′, 5′-AAGGAAGTTTACATTATGCACCTGCGAGGA-3′/5′-AACCACCACCACCGTTCAGTGCACTTTGGTGAGC-3′, 5′-AAGGAAGTTTATGGTTCTAGTATCTATAAG-3′/5′-AACCACCACCACCGTGTTTGGAACCAGTTATTCG-3′, and 5′-AAGGAAGTTTAAGCGAATTCCCAACAGGTG-3′/5′-AACCACCACCACCGTAGACGGGGAAGTGTCAATC-3′. The BSMV-mediated gene silencing in wheat leaves was performed as described by Zhi et al. (2020) [52]. About 15 days after BSMV infection, the newly grown upper leaves with virus symptoms were collected and subjected to inoculation with *B.g. tritici* strain E09 conidia. About 72 h post-*B.g. tritici* inoculation, leaf segments were fixed with ethanol: acetic acid solution (1:1, *v*/*v*) and kept in the destaining solution (lactic acid: glycerol: water, 1:1:1, *v*/*v*/*v*). Before mounting for microscopy, *B.g. tritici*-infected leaves were stained with 0.1% (*w*/*v*) Coomassie Brilliant Blue R250 to visualize the fungal epiphytic structure, as reported previously [52].

### 4.4. Single-Cell Transient Gene Silencing and Overexpression Assay

Antisense fragments of *TaCAMTA2*, *TaCAMTA3*, *TaSARD1*, and *TaEDS1* were, respectively, amplified using the primers 5′-GGGGACAAGTTTGTACAAAAAAGCAGGCTTCTACCATCATTAGCACTTGG-3′/5′-GGGGACCACTTTGTACAAGAAAGCTGGGTCCACTTTTGGAATTACATTC-3′, 5′-GGGGACAAGTTTGTACAAAAAAGCAGGCTTCCATTATGCACCTGCGAGGA-3′/5′-GGGGACCACTTTGTACAAGAAAGCTGGGTCTCAGTGCACTTTGGTGAGC-3′, 5′-GGGGACAAGTTTGTACAAAAAAGCAGGCTTCTGGTTCTAGTATCTATAAG-3′/5′-GGGGACCACTTTGTACAAGAAAGCTGGGTCGTTTGGAACCAGTTATTCG-3′, and 5′-GGGGACAAGTTTGTACAAAAAAGCAGGCTTCAGCGAATTCCCAACAGGTG-3′/5′-GGGGACCACTTTGTACAAGAAAGCTGGGTCAGACGGGGAAGTGTCAATC-3′, and cloned into the pIPKb007 vector using a Gateway cloning system to create the TIGS-*TaCAMTA2*, TIGS-*TaCAMTA3*, TIGS-*TaSARD1*, and TIGS-*TaEDS1* constructs. The coding regions of *TaCAMTA2-4A*, *TaCAMTA2-4B*, *TaCAMTA2-4D*, *TaCAMTA3-2A*, *TaCAMTA3-2B*, *TaCAMTA3-2D*, *TaSARD1.1-6A*, *TaSARD1.1-6B*, *TaSARD1.1-6D*, *TaSARD1.2-6A*, *TaSARD1.2-6B*, *TaSARD1.2-6D*, *TaEDS1-5A*, *TaEDS1-5B*, and *TaEDS1-5D* were, respectively, amplified using the primers 5′-GGGGACAAGTTTGTACAAAAAAGCAGGCTTCATGGCCGAGGGCCGGCGCTAC-3′/5′-GGGGACCACTTTGTACAAGAAAGCTGGGTCCTAGAAATAGCCCGGCAACG-3′, 5′-GGGGACAAGTTTGTACAAAAAAGCAGGCTTCATGGCCGAGGGCCGGCGCTAC-3′/5′-GGGGACCACTTTGTACAAGAAAGCT GGGTCCTAGAAATAGCCAGGCAACG-3′, 5′-GGGGACAAGTTTGTACAAAAAAGCAGGCTTCATGGCCGAGGGCCGGCGCTAC-3′/5′-GGGGACCACTTTGTACAAGAAAGCTGGGTCCTAGAAATAGCCCGGCAACG-3′, 5′-GGGGACAAGTTTGTACAAAAAAGCAGGCTTCATGGCGGAGATGCACAAGTAC-3′/5′-GGGGACCACTTTGTACAAGAAAGCTGGGTCTCACAAAATATTGGACATCG-3′, 5′-GGGGACAAGTTTGTACAAAAAAGCAGGCTTCATGGCGGAGATGCACAAGTAC-3′/5′-GGGGACCACTTTGTACAAGAAAGCTGGGTCTCACAAAACAGTGGACATCG-3′, 5′-GGGGACAAGTTTGTACAAAAAAGCAGGCTTCATGGCGGAGATGCACAAGTAC-3′/5′-GGGGACCACTTTGTACAAGAAAGCTGGGTCTCACAAAATAGTGGACATCG-3′, 5′-GGGGACAAGTTTGTACAAAAAAGCAGGCTTCATGTCTGTGCGAAGGCCGCG-3′/5′-GGGGACCACTTTGTACAAGAAAGCTGGGTCTTAATCAACTTGATCCCAAC-3′, 5′-GGGGACAAGTTTGTACAAAAAAGCAGGCTTCATGTCTGTGCGAAGGCCGCG-3′/5′-GGGGACCACTTTGTACAAGAAAGCTGGGTCTTAATCAACTTGATCCCAAC-3′, 5′-GGGGACAAGTTTGTACAAAAAAGCAGGCTTCATGTCTGTGCGAAGGCCGCG-3′/5′-GGGGACCACTTTGTACAAGAAAGCTGGGTCTTAATCAACTTGATCCCAAC-3′, 5′-GGGGACAAGTTTGTACAAAAAAGCAGGCTTCATGTCGGTGCGAAGGCCCCG-3′/5′-GGGGACCACTTTGTACAAGAAAGCTGGGTCTTAATCAACTTGATCCCAAC-3′, 5′-GGGGACAAGTTTGTACAAAAAAGCAGGCTTCATGTCGGTGCGAAGGCCACG-3′/5′-GGGGACCACTTTGTACAAGAAAGCTGGGTCTTAATCAACTTGATCCCAAC-3′, 5′-GGGGACAAGTTTGTACAAAAAAGCAGGCTTCATGCCGATGGACACCCCGCC-3′/5′-GGGGACCACTTTGTACAAGAAAGCTGGGTCTTACGAAGGCACAAGTCTCGC-3′, 5′-GGGGACAAGTTTGTACAAAAAAGCAGGCTTCATGCCGATGGACACCCCGCC-3′/5′-GGGGACCACTTTGTACAAGAAAGCTGGGTCTTACGAAGGCACAAGTCTCGC-3′, and 5′-GGGGACAAGTTTGTACAAAAAAGCAGGCTTCATGCCGATGGACACCCCGCC-3′/5′-GGGGACCACTTTGTACAAGAAAGCTGGGTCTTACGAAGGCACAAGTCTCGC-3′, and cloned into the pIPKb001 vector. The single-cell transient gene silencing and expression were conducted essentially as described (Zhi et al., 2020) [52]. Briefly, the GUS reporter vector was co-delivered (1:1 molar ratio) with pIPKb001 or pIPKb007 constructs into the wheat epidermal cell through the particle inflow gun (Bio-Rad). After inoculation with *B.g. tritici* strain E09 conidia, the leaf segments were stained for GUS activity 48 h post-*B.g. tritici* inoculation. Before mounting for microscopic analysis, the leaves were stained with 0.1% (*w*/*v*) Coomassie Brilliant Blue R250 to visualize the fungal epiphytic structure.

## Data Availability

Data presented here are available on request from correspondence.
